# Sr-substituted bone cements direct mesenchymal stem cells, osteoblasts and osteoclasts fate

**DOI:** 10.1371/journal.pone.0172100

**Published:** 2017-02-14

**Authors:** Monica Montesi, Silvia Panseri, Massimiliano Dapporto, Anna Tampieri, Simone Sprio

**Affiliations:** Institute of Science and Technology for Ceramics, National Research Council, Faenza, Ravenna, Italy; Kyungpook National University School of Medicine, REPUBLIC OF KOREA

## Abstract

Strontium-substituted apatitic bone cements enriched with sodium alginate were developed as a potential modulator of bone cells fate. The biological impact of the bone cement were investigated *in vitro* through the study of the effect of the nanostructured apatitic composition and the doping of strontium on mesenchymal stem cells, pre-osteoblasts and osteoclasts behaviours. Up to 14 days of culture the bone cells viability, proliferation, morphology and gene expression profiles were evaluated. The results showed that different concentrations of strontium were able to evoke a cell-specific response, in fact an inductive effect on mesenchymal stem cells differentiation and pre-osteoblasts proliferation and an inhibitory effect on osteoclasts activity were observed. Moreover, the apatitic structure of the cements provided a biomimetic environment suitable for bone cells growth. Therefore, the combination of biological features of this bone cement makes it as promising biomaterials for tissue regeneration.

## Introduction

Osteoporosis is a global public health problem currently affecting nearly 50 million of individuals in the industrialized countries and representing a major public health burden nowadays and for the foreseeable future [1]. It is characterized by an imbalance in bone remodelling process that leads to progressive loss of bone mass and bone microarchitecture, thus increasing the fracture risk [2]. Osteoporotic fractures have serious direct consequences on the patient quality of life (e.g. decreased functional mobility) and indirect consequences on the whole society (e.g. increase request of professional home-care services). The resulting socio-economic cost is estimated at about 40 billion of euro per year in the European Union [3, 4].

Currently, together with the lifestyle recommendations for reducing osteoporotic fracture risk, the strategies to treat or limit the progress of the osteoporosis include the surgical management of osteoporotic fractures by minimally invasive techniques with injectable methyl methacrylate cements [5], pharmacological therapies with bisphosphonates, hormonal therapy, antagonists of the Wnt signaling pathways and anti-resorptive drugs and molecules acting on calcium-sensing receptors [6–8]. However, all these strategies confine the problem without resolving it. For example, the oral administration of bisphosphonates has several severe side effects and the current injectable biomaterials addressed to bone healing are still unable to exhibit adequate bioactivity and osteoconductivity, associated with the adequate mechanical performance to withstand the early biomechanical loads [9–12].

In this respect, the development of injectable biomaterials provided with bioactivity, bioresorbability as well as the ability to be implanted by minimal invasive surgery and self-harden *in vivo*, stabilizing even complex-shape bone fractures is highly demanded. Nowadays, a growing interest on the synthesis and development of calcium phosphate bone cements (CPCs) is demonstrated in literature, especially due to their excellent bioactivity deriving from the chemical similarity to the inorganic part of bone [13–15].

Among the various approaches reported for the synthesis of CPCs, the one based on the hydrolysis and transformation of α-Ca_3_(PO_4_)_2_ (αTCP) into nanostructured calcium-deficient hydroxyapatite (CD-HA) particles is particularly valued [9]. Moreover, the possibility to endow CD-HA structure of bioactive ions increased the possible application of this bone cement. In particular, the CD-HA doping with strontium ions is particularly effective against osteoporotic bone weakening [16, 17]. It was found that Sr^2+^ enhances the proliferation and differentiation of osteoprogenitor cells into bone-forming osteoblasts, with mechanisms probably involving membrane-bound calcium sensing receptor (CaSR) and Wnt/β-catenin signalling [18, 19]. Moreover, Sr^2+^ decreases osteoclastogenesis and the resorbing activity of mature osteoclasts [20, 21].

In the present work, Sr-doped hydroxyapatite (HA) bone cements were prepared by mixing Sr-substituted αTCP phases with setting solutions enriched with sodium alginate, as already described and characterized in our previous work [22], and for the first time were deeper biological evaluates *in vitro*, to investigate its biological effect on mouse mesenchymal stem cells (MSCs), pre-osteoblasts (OBs) and osteoclasts (OCLs).

## Materials and methods

### Sr-BCs synthesis

Sr-doped HA cements were prepared as reported in our previous work [22]. Briefly, Sr-substituted αTCP powders with different strontium content (i.e. Sr/(Ca+Sr) = 0, 2, 5 mol%, henceforth coded as bone cement (BC), Sr2%-BC and Sr5%-BC respectively), were synthesized by solid state reaction at 1400°C for 1 hour of mixtures of calcium carbonate (CaCO_3_, Carlo Erba, Italy), calcium hydrogen phosphate (CaHPO_4_, Sigma Aldrich) and strontium carbonate (SrCO_3_, Carlo Erba, Italy). After rapid quenching, the powders were milled by planetary mono mill (Pulverisette 6 classic line, Fritsch, Germany) at 400 rpm for 50 minutes with 5 mm diameter zirconia balls. Aqueous solutions containing 5 wt% of Na_2_HPO_4_ (Fluka) and 2 wt% of sodium alginate (Alginic Acid Sodium Salt from Brown Algae, Sigma Aldrich) were mixed with the powders by using a liquid-to-powder ratio equals to 0.48 ml/g. Solid and liquid components were treated with γ irradiation (25 kGy) and autoclaving (121°C for 20 minutes), respectively, and then mixed together by using a commercial Bone Cement Delivery System equipment (P-system, Medmix, Switzerland).

Cylindrical cement specimens for *in vitro* tests (diameter = 10 mm; height = 2 mm) were obtained after 72 hours of dry incubation at 37°C and washing in ethanol 70% for 20 min followed by three washes in 1x PBS for 10 min each. The discs were then dried and sterilized by UV irradiation for 30 min per side under laminar flow hood and finally preconditioned for 72 hours in standard cell culture medium.

### Ion concentration measurements

**Fig 1:**
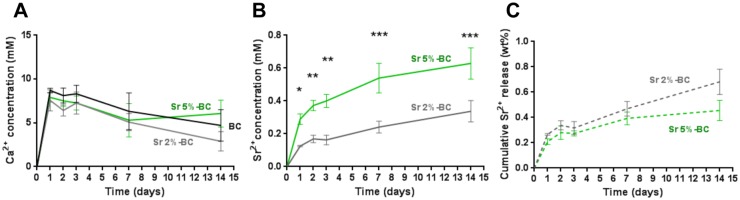
Ion concentration. Cumulative Ca^2+^ (A) and Sr^2+^ (B) ion release concentrations from the cements in Dulbecco's modified Eagle's medium and resulting ion concentration (in weight %) in respect to the initial amount of Sr^2+^ in the precursor powders (C). (*p≤0.05; **p≤0.01, ***p≤0.001).

The elemental composition of the cement precursors as well as the release of calcium and strontium ions in cell culture medium were analysed by Optical Emission Spectrometry (ICP-OES, Liberty 200, Varian, Clayton South, Australia). In particular, 20 mg of precursor powders were dissolved into 2 ml of nitric acid (HNO_3_, Sigma Aldrich), followed by dilution to 100 ml with bi-distilled water. Then, the release of calcium and strontium ions was evaluated by submerging disk-shaped samples (diameter = 10 mm; height = 2 mm) in Dulbecco's modified Eagle's medium (DMEM/F12 GlutaMAX (Gibco, Carlsbad, CA) supplemented with 10% Fetal Bovine Serum and 1% Penicillin-Streptomycin (PAA, Pasching, Austria) and aged at 37°C and 5% CO_2_. At each time point (after 1, 2, 3, 7 and 14 days) of incubation the supernatants were completely removed from the sample and collected for ion quantification (in mM): the supernatants were diluted in 0.5 ml HNO_3_ and 8.5 ml bidistilled water. Three specimens for each precursor powder and cement formulation were tested. The amount of Sr^2+^ ions (in weight) released at each time point was also referred to the correspondent amount in the precursor powder (in weight) (Fig 1C).

### Cell culture

Mouse mesenchymal stem cells (C57BL/6) (MSCs), purchased from Invitrogen (Carlsbad, CA), were cultured in DMEM/F12 GlutaMAX (Gibco, Carlsbad, CA) supplemented with 10% Fetal Bovine Serum (FBS) and 1% Penicillin-Streptomycin (100 U/ml-100 μg/ml); PAA, Pasching, Austria). For the experiments, cells were plated at 1.5×10^4^/cement and cultured for up to 14 days in αMEM GlutaMAX (Gibco) supplemented with 10% FBS and 1% Penicillin-Streptomycin and osteogenic factors (10 mM β-glycerophaspate, 10^−7^ M dexamethasone, 50 μg/ml ascorbic acid).

Mouse pre-osteoblast cell line MC3T3-E1 Subclone 14 (OBs), obtained from ATCC cell bank (Manassas, VA, USA), was used as a model of osteoblasts [23]. 1.5×10^4^/cement of OBs were cultured in αMEM containing ribonucleosides, deoxyribonucleosides (GIBCO), and L-glutamine, 10% FBS and 1% Penicillin-Streptomycin and osteogenic factors (10 mM β-glycerophaspate, 50 μg/ml ascorbic acid).

The RAW 264.7 cell line (ATCC) (OCLs), an established mouse monocyte cell line used as a model of osteoclast formation *in vitro* [24], was cultured in DMEM/F12 GlutaMAX supplemented with 10% FBS and 1% Penicillin-Streptomycin. To initiate the cell differentiation, 25 ng/mL soluble murine Receptor Activator for Nuclear Factor kB Ligand (RANKL, Sigma-Aldrich, Dorset, UK) were added. For the experiments, cells were plated at 3.0×10^4^/cement and cultured for up to 14 days.

Sr-BCs and BC discs, sterilized and preconditioned as above derived, were placed one per well in a 24-well plate and a drop of 50 μl, containing cell suspension, was seeded on the centre of the upper discs surface allowing cell attachment for 30 min in the incubator, before adding 1.5 ml of cell culture medium into each well. The cell medium was changed every 3 days. All cell-handling procedures were performed in a sterile laminar flow hood. All cell culture incubation steps were performed at 37°C with 5% CO_2_ and controlled humidity.

### Cell viability and proliferation

The viability and proliferation of the MSCs and OBs were measured by evaluating metabolically active cells. The MTT reagent (3-(4,5-dimethylthiazol-2-yl)-2,5-diphenyltetrazolium bromide) is reduced to formazan dye in metabolically active cells. The formazan production can be observed at λ_max_ of 570 nm, using a Multiskan FC Microplate Photometer (Thermo Scientific), and the absorbance is directly proportional to the number of metabolically active cells.

The reagent was prepared at 5 mg/mL in 1x PBS. Cell were incubated with the MTT reagent 1:10 for 2 h at 37°C. Medium was collected and cells incubated with dimethyl sulfoxide for 15 min. In this assay, the mean values of absorbance were determined. Tree biologically independent samples were analysed at day 1, 2, 3, 7, and 14 and the percentage of viability with respect to that of cells cultured on BCs, used as control group, was shown.

### Cell morphology evaluation

#### Actin filaments staining

After 3 days of culture, samples were washed in 1x PBS for 5 min and fixed in 4% (w/v) paraformaldehyde for 15 min. Permeabilization was performed with 1x PBS with 0.1% (v/v) Triton X-100 for 5 min. FITC conjugated phalloidin (Invitrogen) 38 nM in 1x PBS was added for 20 min at room temperature in the dark. For nuclear staining the cells were incubated with DAPI 300 nM (Invitrogen) for 5 min. Images were acquired by an Inverted Ti-E fluorescence microscope (Nikon). The analysis was performed for all the cements tested, but only one representative image for each cell type grown on BC samples has been reported.

#### Scanning Electron Microscopy (SEM) analysis

After 3 days of culture, Sr-BCs and BCs samples were washed with 0.1 M sodium cacodylate buffer pH 7.4 and fixed in 2.5% glutaraldehyde in 0.1 M sodium cacodylate buffer pH 7.4 for 2 h at 4°C, washed in 0.1 M sodium cacodylate buffer pH 7.4 and dehydrated in a graded series of ethanol (30%, 50%, 70%, 90% and 100% for 10 min/each). Dehydrated samples were sputter-coated with gold and observed using Stereoscan 360 SEM (Cambridge Instruments, UK). The analysis was performed for all the cements tested, but one representative image for each cell type grown on BC samples has been reported.

### Quantitative real-time polymerase chain reaction (qPCR)

After 7 and 14 days, cells grown on the cements were lysed and total RNA extraction was performed by use of the Tri Reagent, followed by the Direct-zol^™^ RNA MiniPrep kit (Euroclone, Milano, Italy) according to manufacturer's instructions. RNA integrity was analysed by native agarose gel electrophoresis and quantification performed by the Qubit^®^ 2.0 Fluorometer together with the Qubit^®^ RNA BR assay kit, following manufacturer's instructions (Invitrogen). Total RNA (500 ng) was reverse transcribed to cDNA using the High-Capacity cDNA Reverse Transcription Kit, according to manufacturer's instructions (Life Technologies, Carlsbad, CA). Quantification of the gene expression was performed by use the StepOne^™^ Real-Time PCR System (Applied Biosystems, Foster City, CA, USA). The target genes evaluated for MSCs were: runt-related transcription factor 2 (Runx2, Mm 00501580), alkaline phosphatase (ALP, Mm00475834); target genes for MC3T3-E1 evaluation were: Osterix (Mm 04209856), osteocalcin (Bglap, Mm 00649782) and integrin-binding sialoprotein (IBSP, Mm 00492555); for the quantification of the osteoclast activity the target genes evaluated were: osteoclast-associated immunoglobulin-like receptor (OSCAR, Mm00558665), integrin beta-3 (Itgβ3, Mm00443980) and cathepsin K (CtsK, Mm00484039).

The housekeeping gene used for all the assays was glyceraldehyde 3-phosphate dehydrogenase (GAPDH, Mm99999915) (Life Technologies). Three biologically independent samples were analysed. BC’s error bars reflect one standard deviation from the mean of 3 technical replicates as described [25, 26]. Data were collected using the StepOne Software (v.2.2.2) and relative quantification was performed using the comparative threshold (Ct) method (ΔΔCt), where relative gene expression level equals 2^-ΔΔCt^ [27]; BC sample was used as calibrator.

### Statistical analysis

Results were expressed as mean ± standard error of the mean (SEM) plotted on graph. Analysis of the effect of the cements on cell culture and of the ion release in culture medium was made by two-way analysis of variance (ANOVA), followed by Bonferroni’s post hoc test for biological results and by Sidak's multiple comparisons test for chemical tests. Statistical analyses were performed by the GraphPad Prism software (version 6.0).

## Results

### Strontium and calcium release

Upon immersion of the cement samples, a slight acidification of the culture medium was observed, as a colour change towards yellow occurred, especially for BC formulation. In this respect, if compared with the pH of physiological fluids [28], after 7 days, a ΔpH = -0.4, -0.36 and -0.13 was detected for BC, Sr2%-BC and Sr5%-BC, respectively. The concentrations of Ca^2+^ and Sr^2+^ ions, net of the amount of ions leached out during preconditioning, are shown in Fig 1A. Significant calcium depletion over time was registered from day 3 to day 14 (p<0.05) (Fig 1A), without difference among the cements. Conversely, the Sr^2+^ release steadily was increased for all the samples up to 14 days; Sr5%-BC exhibited a higher strontium release, if compared with Sr2%-BC, with a decreased release rate after 1 day. After 14 days Sr5%-BC exhibited a Sr^2+^ concentration of 0.63 mM, two times higher than for Sr2%-BC (0.34 mM) (Fig 1B). Moreover, the overall amount of strontium released up to 14 days was very limited (in respect to the amount of ion detected in the precursor powders) and without plateaus (Fig 1C, 0.68 wt% for Sr2%-BC and 0.48 wt% for Sr5%-BC), suggesting a potential capability of the cement to further ion release.

### Biological effect of Sr-BCs on MSCs

**Fig 2:**
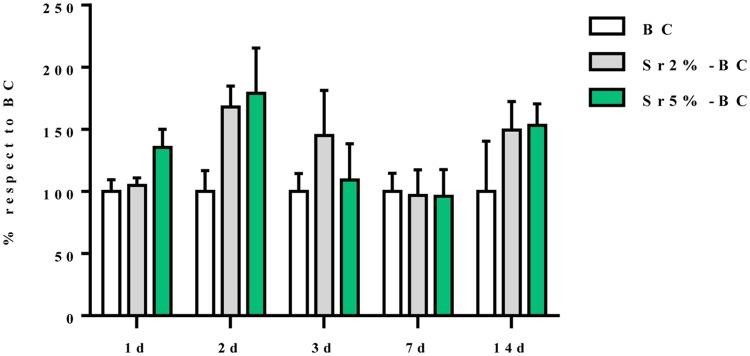
MSCs viability. Percentage of MSCs viability grown in Sr2% and 5%-BCs compared to the cells grown in BC. Statistical analysis showed no differences among the samples tested and over the experimental time points.

The results showed that the presence of the Sr^2+^ increased the bioactivity of this cement. Concerning the MSCs viability and proliferation, measured by evaluating metabolically active cells (MTT assay), no significant difference existed between the effect of Sr2%-BC and Sr5%-BC and no significant differences existed also in cell viability over the time of culture (Fig 2). However, it was possible to observe a weak decreasing at day 3 and day 7. That variability can be attributed to the imbalance of ions induces by the presence of the cements in a static culture condition that, however, do not compromise the MSCs viability.

**Fig 3:**
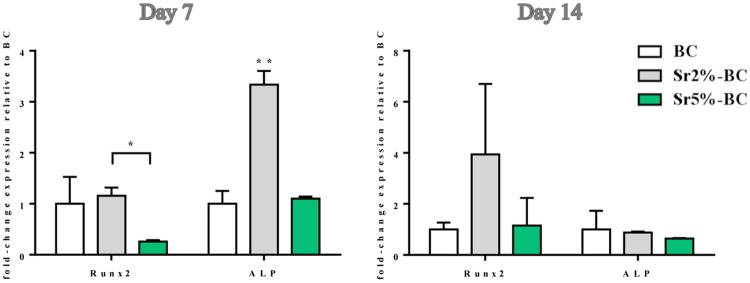
MSCs gene expression analysis. Relative quantification of gene expression after 7 and 14 days of MSCs grown on Sr-BCs. The graph showed the fold change expression of RUNX2 and ALP, relative to the expression of the MSCs grown on BCs, used as a control (*p≤0.05; **p≤0.01).

To evaluate the inductive effects on the gene profile related to the osteogenic differentiation, the expression of Runx2 and ALP, considered the principal markers of osteoblast commitment, has been quantified. MSCs, grown for 7 days on Sr2%-BC, displayed a significant increase in mRNA level of both the genes compared to the Sr5%-BC (Runx2 p≤0.05 and ALP p≤0.01) (Fig 3).

**Fig 4:**
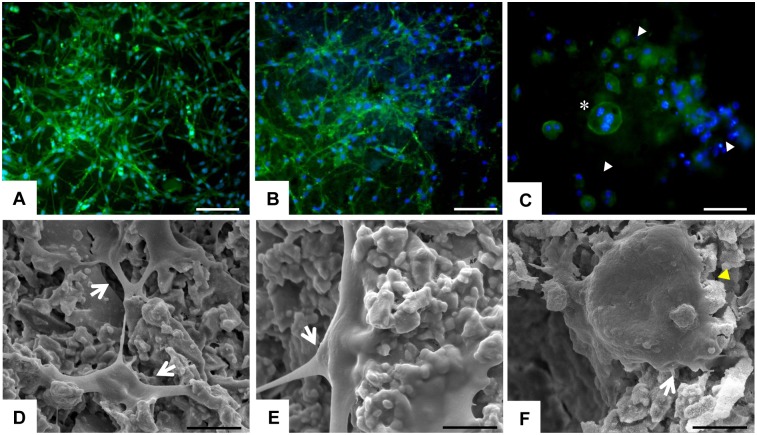
Cells morphology. The upper level on the panel showed phalloidin staining: in green the cytoplasm of the cells and in blue the nuclei. At 3d, MSCs (A) and OBs (B) were well spread on BC surface exhibiting their characteristic morphology without any difference among each sample; scale bars 200 μm. Image (C) showed a big multinucleate OCLs (*) and groups of undifferentiated monocytes (white arrows); scale bar 50 μm. On the lower level of the panel SEM images are showed: D and E showed the cytoplasmic extension (white arrows) of MSCs and OBs, respectively; scale bars 5 μm. (F) One OCLs grown on BCs surface, exhibiting the typical apical-basal polarised resorbing morphology (yellow arrow); scale bar 10 μm.

The cell morphological analysis (Fig 4A) showed that MSCs were well spread and appeared to be homogenously distributed on the surface of all the tested cements, without any remarkable differences among them. After 3 days the cement surface of all the samples was nearly completely covered by the MSCs that exhibited their characteristic morphology and showed cytoplasmic extensions connecting them to each other and to the biomaterial surface, indicating the good cytocompatibility of the proposed cements (Fig 4D). Since there are no differences in cell morphology between the three different cements, only one representative image for each cell type was reported in Fig 4.

### Biological effect of Sr-BCs on OBs

**Fig 5:**
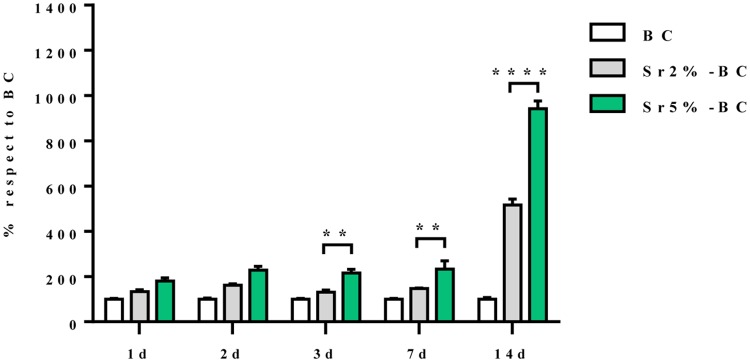
OBs viability. The viability of OBs grown in Sr2% and 5%-BCs were increased compared to the cells grown in BC (Two-way Anova p≤0.0001). Statistical analysis shown significant differences between Sr2%-BC and Sr5%-BC after 3, 7 and 14 days of culture (**p≤0.01; ****p≤0.0001).

**Fig 6:**
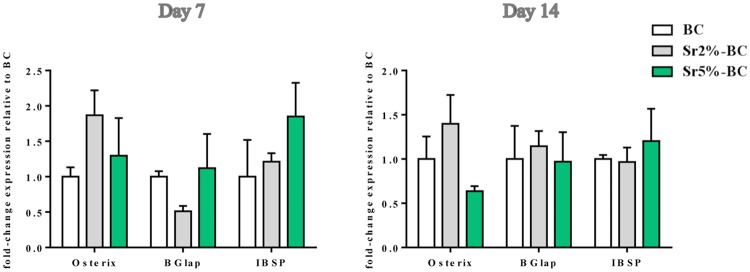
OBs gene expression analysis. Relative quantification of gene expression after 7 and 14 days of OBs grown on Sr-BCs. The graph showed the fold change expression of Osterix, Bglap and IBSP, relative to the expression of the OBs grown on BCs, used as a control.

OBs viability, assessed by MTT analysis, showed an increase over the experimental time. Sr5%-BC induced a significant higher level of proliferation compared to Sr2%-BC, after 3 days, 7 days (p≤0.01) and 14 days (p≤0.0001) of culture. Moreover significant differences were observed between BC and both Sr substituted BC at all the time points (p≤0.0001) (Fig 5). Instead, the analysis of gene expression profile showed no significant difference directly ascribable to Sr^2+^ in the induction of osteoblast related genes (i.e. Osterix, BGlap and IBSP) among all the BCs tested (Fig 6).

The study of cell morphology, through the cytoskeletal actin filaments analysis and the SEM evaluation, showed OBs exhibiting the typical morphology and strictly interacting with the BCs surface without any differences among all the tested cements. In Fig 4B and 4E it is possible to observe stretched cellular pseudopodia anchoring the cement surface or connecting the contiguous cells.

### Biological effect of Sr-BCs on OCLs

**Fig 7:**
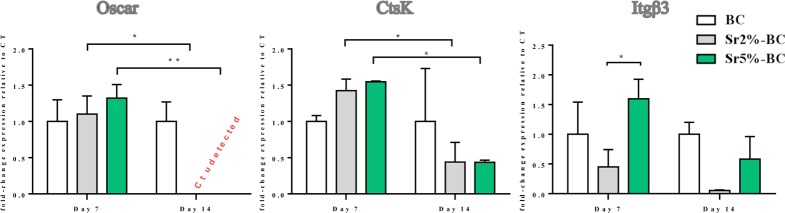
OCLs gene expression analysis. Relative quantification of gene expression after 7 and 14 days of OCLs grown on Sr-BCs. The graph showed the fold change expression of Oscar, CtsK and Itgβ3 relative to the expression of the OCLs grown on BCs, used as a control. Statistical differences exist for Oscar and CtsK expression between day 7 and day 14 of culture (*p≤0.05; ** p≤ 0.01).

Gene expression analysis (Fig 7) showed a significant decrease occurring over the time of Oscar and CtsK mRNA level, genes involved in the mechanism of OCLs differentiation and bioactivity. The Itgβ3 mRNA level showed a difference between Sr2%-BCs and Sr5%-BC after 7 days (p≤0.05), that became negligible after 14 days of culture.

Actin staining showed several undifferentiated monocytes and few mature OCLs with the typical podosome belts, an actin-rich structure, which appeared as prominent rings encasing multiple nuclei. SEM images showed the details of one mature OCL grown on BC surface exhibiting the basoapically polarized morphology and the osteoclast/mineralized substratum interface typically of the resorption process (Fig 4C and 4F).

## Discussion

Biomaterials play a crucial role in personalized medicine due to their unique features in tuning cell behaviour. The injectable biomaterials represent an excellent strategy to improve the interaction of the material itself with the surrounding tissues in osteoporotic patients [29–31]. In the present study, it has been demonstrated that novel injectable strontium-doped apatitic bone cements [22], influenced the viability, morphology and gene expression profiling of bone cells. A detailed morphological analysis showed MSCs and OBs well attached and spread on all the cement surfaces with their characteristic morphology. It was possible to observe stretched cellular pseudopodia anchoring the cement surface or connecting the contiguous cells as index of high level of cell-cell and cell/material interaction.

The qualitative OCLs morphological analysis confirmed the presence of few mature osteoclasts on all the BCs surface, surrounded by undifferentiated monocytes. This was considered as a marker of OCLs integration and a good indicator of the grade of the mimicry of the cement apatite structure [32, 33]. These findings suggested that the complex phenomena occurring during the cell/material interaction could be directly related to the biomimetic apatitic structure of the bone cements.

Although the presence of Sr^2+^ seemed to not induce modification in cell morphology, Sr^2+^ conferred to the BCs biological activity able to modulate the bone cell behaviour due to its already demonstrated anabolic activity on MSCs and OBs, and inhibitory effect on OCLs [19, 34–36].

Currently, one major treatment of osteoporosis is the systemic administration of strontium ranelate. However, the use of this drug has been associated with adverse side effects such as venous thrombosis, cutaneous hypersensitivity, chronic renal failure, and other, and its bioavailability is very low by oral administration (<20%) [37, 38]. Consequently, sustained local release of Sr^2+^ ions from biomaterials could be preferable to the systemic administration.

In this work, the effect of Sr^2+^ substitution on the degradation properties of Sr-BCs, which consist in the releasing of Sr^2+^ and Ca^2+^ ions, up to 14 days of immersion in complex media able to replicate the *in vivo* fluids was studied. Generally, the introduction of strontium in the apatite structure is associated to an increased solubility of the cements, lead to an increase of ions released, due to its lattice expansion [39, 40]. It has been already proved that modification of ions concentration (e.g. Calcium) in the cell culture medium can even greatly affect both cell proliferation and differentiation [41, 42]. It was observed that αTCP-based cements generally induce a slight acidification and a depletion of Ca^2+^ concentration in the immersion medium possibly due to the progressive formation of apatite crystals [40]. In our system, a Ca^2+^ depletion in the culture medium overtime was also observed, while the introduction of strontium seems to attenuate the pH fluctuation; conversely, the Sr^2+^ release increased overtime, possibly attenuating the reported inhibiting effect of Ca^2+^ depletion on cell behaviour [43].

It should be noted that, after 1 day, the strontium release of Sr2%-BC (0.125 mM) was really close to the reported circulating strontium concentrations in osteoporotic patients treated with strontium ranelate orally administrated at a dose of 2 g daily (0.12 mM) [44–46]. This result makes this cement a good candidate for the treatment of osteoporotic bone fractures and defects, as a local reservoir of Sr^2+^ ions, to avoid the side effects related to the high oral dose treatments administered to compensate the relatively low bioavailability of the drugs.

*In vitro* results suggested that the Sr^2+^ concentration released from the Sr-BCs is able to induce specific cellular response depending on the bone cell types, with totally absence of cytotoxicity also at the highest concentration detected (0.628 mM). Confirming the already known biological role of the Sr^2+^ [47], here it has been shown an effect of the released Sr^2+^ on the induction of MSCs osteogenesis-related gene (Runx2 and ALP). Interestingly, the activity of 0.24 mM of Sr^2+^, released from Sr2%-BC after 7 days, was higher compared to the effect of 0.54 mM of Sr^2+^ released from Sr5%-BC. The presence of Sr^2+^ had a different behaviour on precursor cells committed to the bone lineage (OBs). In fact, Sr^2+^ evoked a dose-dependent inductive effect on the proliferative process of OBs, but it seemed to be not directly involved in the expression of the mature osteoblast related genes. Several *in vitro* studies demonstrated a wide range of different responses induced by Sr^2+^ in term of differentiation and proliferation, depending on the cell type, time and dose of Sr^2+^ administration [47–50]. It seems reasonable to hypothesize that the variability of biological effects observed could be explained by the differences in the cell models, pre-osteoblasts and mesenchymal stem cells, as Sr^2+^ exerted a defined role that probably depended on the different commitment stage of the cells. The *in vitro* study confirmed also that the Sr^2+^ released from Sr-BCs exerts its inhibitory effect on the expression of the principal genes involved in OCLs activity (Oscar and CtsK) [51–53]. Although Itgβ3 mRNA level seems to follow the same trend of decreasing over the time of culture, no statistically difference was observed, probably due to the proved role of integrin β3 in the interaction with apatitic structure [54]. In fact, Itgβ3 is considered the principal receptor in osteoclasts mechanisms involved in adhesion to the extracellular matrix, motility and activity. Therefore, the observed absence of Itgβ3 down-regulation mediated by Sr^2+^ is in accordance with the high level of OCLs interaction with the apatitic structure of the BCs, as demonstrated by the morphological analysis.

## Conclusion

In this work, strontium-doped apatitic bone cements doped with tailored amounts of strontium and enriched with sodium alginate were biological characterized. The three different cell types studied exhibited different behaviour, depending on the Sr^2+^ doping amount. In this respect, it was possible to conclude that the Sr^2+^ is able to promote: i) an inductive effect on MCS osteogenic gene expression, especially at 2 mol% concentration; ii) a dose-dependent inductive effect on OBs proliferation and iii) an inhibitory effect on OCLs activity. Thanks to the combination of the biomimetic features of the apatitic bone cement structure with the biological effect of Sr^2+^, such cements were able to evoke a specific cellular response, thus exhibiting promising features to assist new bone formation, particularly relevant in the case of bone turnover impairment due to metabolic diseases, such as osteoporosis.
